# Natural Killer Cell Subsets and IL-2, IL-15, and IL-18 Genes Expressions in Chronic Kidney Allograft Dysfunction and Graft Function in Kidney Allograft Recipients

**Published:** 2016-11-01

**Authors:** S. Assadiasl, A. Sepanjnia, B. Aghili, M. Nafar, P. Ahmadpoor, F. Pourrezagholi, M. Parvin, A. Shahlaee, M. H. Nicknam, A. Amirzargar

**Affiliations:** 1 *Department of Immunology, School of Medicine, Tehran University of Medical Sciences, Tehran, Iran*; 2 *Chronic Kidney Disease Research Center, Labbafinejad Hospital, Shahid Beheshti University of Medical Sciences, Tehran, Iran*; 3 *Department of Pathology, Labbafinejad Hospital, Shahid Beheshti University of Medical Sciences, Tehran, Iran*; 4 *Molecular Immunology Research Center, Tehran University of Medical Sciences, Tehran, Iran*

**Keywords:** Chronic allograft dysfunction, Natural killer cells, Interleukin gene expression

## Abstract

**Background:**

While acute rejection and early graft loss rates have decreased substantially over the past four decades, progressive chronic allograft dysfunction (CAD) still remains a common cause of late graft loss in kidney transplant recipients.

**Objective:**

This study was conducted to investigate the percentage of natural killer (NK) cell subsets and IL-2, 15 and 18 genes expression in two groups of CAD and well-function graft (WFG) recipients.

**Methods:**

30 renal allograft recipients with biopsy-proven interstitial fibrosis/tubular atrophy (IF/TA) and impaired renal function, and 30 sex- and age-matched WFG patients were enrolled in this study. The percentage of NK cell subsets including NK CD56^bright^ and NK CD56^dim^ cells were determined by flowcytometry; IL-2, IL-15, and IL-18 genes expressions were assessed by real-time PCR.

**Results:**

Compared to WFG patients, there was a significant (p<0.05) increase in the percentage of NK CD56^bright^ cells in CAD patients. However, the difference in percentage of NK CD56^dim^ cells or CD56^dim^/CD56^bright^ ratio between the studied groups was not significant. In addition, IL-2, 15 and 18 genes expressions were almost similar in CAD and WFG patients.

**Conclusion:**

We found higher percentages of NK CD56^bright^ subset in kidney transplant recipients with CAD without considerable changes in related cytokines’ gene expression, suggesting a possible defect of NK cells maturation in these patients.

## INTRODUCTION

Kidney allograft transplantation is the treatment of choice for end-stage renal diseases. Both antigen-dependent (*e.g.*, HLA mismatch) and antigen-independent risk factors (*e.g.*, ischemia reperfusion injury [IRI]) can result in inflammation, tissue damage, and initiation of graft failure [1]. Many studies in the context of solid organ transplantation suggest a considerable role for innate immunity, particularly natural killer (NK) cells and related cytokines, in allograft damages. However, the exact mechanisms have not yet been fully understood [2].

NK cells phenotypically express CD16 and CD56 in the absence of TCR/CD3 complex. Considering the amount of CD56 expression, NK cells are classified as CD56^bright^ and CD56^dim^ cells, but the expression of CD56 is not steady and changes during different stages of maturation. Mature NK cells express NKG2D, low levels of CD56, and high levels of surface CD16. While around 90% of human peripheral blood NK cells are CD56^dim^ and regarded as the classical cytotoxic NK cell subset, the remaining 10%, the so-called CD56^bright^ subset, display low cytotoxic capacity and produce high levels of IFNγ and TNFα [3].

NKCD56^dim^ cells produce cytolytic molecules, like perforin, granzyme, and granulyzin, to eliminate unfamiliar allograft cells [4]. But, recent studies have shown presence of regulatory NK cell subsets in stable grafts, indicating a unique role for NK cells in maintenance of homeostasis, introducing them as active participants in rejection or acceptance of transplanted organs [5]. NK cells effect on dendritic cells maturation and activation has been investigated in some studies [6, 7]. For instance, it has been shown that early events following kidney transplantation involving NK-DC interaction via KIR-HLA-C immune synapse and NK cells cytolytic activity against donor-derived APCs influence the transplant outcome [8].

Tissue biopsy is the gold-standard diagnostic test for determining chronic allograft damages, but its invasiveness and consequential side effects limit its feasibility in clinic. Therefore, there is a growing tendency to replace biopsy with a battery of tests for biomarkers in peripheral blood and urine. A combination of NK cells percentages and ratio might be an eligible candidate for the new test [9]. In addition, manipulating the number and activity of NK cell subsets to find new immunomodulatory strategies seems to be helpful. 

In this study we investigated NK CD3^neg^ CD16^dim^ CD56^bright^ and CD3^neg^ CD16^bright^ CD56^dim^ subsets among two groups of patients—a group with chronic allograft dysfunction (CAD) with biopsy-proven interstitial fibrosis/tubular atrophy (IF/TA), and another group of recipients with stable graft function (WFG) in order to find any correlations between clinical status and NK cells counts and ratio in their peripheral blood.

## MATERIALS AND METHODS

Patients

Sixty adult renal transplant recipients, who had received kidney allograft between six months and five years prior to our study, were enrolled in this study. They included 30 patients with biopsy-proven CAD and 30 patients with clinically well-function graft (WFG).

The patients with CAD (n=30) had had a progressive deterioration in their allograft function with 15% or more irreversible rise in creatinine within 1–3 months and proteinuria >1 g/24 h [9]. Consequently, they were biopsied where the pathologic lesions (IF/TA) were reported in all of them [10, 11]. 

Those with WFG (n=30) were selected according to their sex, age and time post-transplantation in order to be pair-matched with CAD patients. They were normal in clinical examination and their serum creatinine levels were ≤1 mg/dL, they had proteinuria <0.5 g/24 h and their Cockcroft creatinine clearance was >80 mL/min [9]. They had no registered history of acute rejection episodes. Since these patients presented no deterioration in their graft function, and protocol biopsies are not routine in our centers, no biopsy was taken for this group. None of the enrolled patients had diagnosed with infectious diseases at the sampling time. The protocol conformed to the ethical guidelines of the 1975 Helsinki Declaration and was approved by the Ethics Committee of Tehran University of Medical Sciences. All patients gave written informed consent prior to the inclusion in our study. 

Flowcytometry

For staining of CD3^neg^ CD16^dim^ CD56^bright^ and CD3^neg^ CD16^bright^ CD56^dim^ NK cells, we used FITC anti-human CD16, APC anti-human CD56, and PE anti-human CD3 (eBioscience, San Diego, USA). FITC conjugate mouse IgG1, mouse IgG1 isotype control PE, and mouse IgG1 isotype control APC (eBioscience, San Diego, USA) were also used as isotype negative controls. Flowcytometry was performed by FACSCalibur (BD FacsCalibur Becton Dickinson, USA) instrument and data were analyzed by CellQuest Pro software.

RNA Isolation and Real-Time PCR

RNAs were isolated from PBMC using high pure RNA isolation kit (Roche Diagnostics, Mannheim, Germany) according to manufacturer’s instruction. RNA quality was assessed by NanoDrop1000 spectrophotometer (Thermo Scientific, USA) and samples with A_260_/A_280_ ratio of 1.8–2.2, and A_260_/A_230_ ratio of 2–2.2 were considered “acceptable.” RNA samples reverse transcription to cDNA was performed by transcriptor first strand cDNA synthesis kit (Roche Diagnostics, Mannheim, Germany). cDNA quality was also evaluated by NanoDrop1000 spectrophotometer (Thermo Scientific, USA) and samples with A_260_/A_280_ ratio of 1.7–2 were stored at 70 °C until use.

Gene expression assay was performed by real-time PCR using TaqManprobs and specific primers supplied by ABI (ABI, Applied BiosystemStepOnePlus). The endogenous control was housekeeping gene, β-actin. Relative gene expression was calculated by standard curve method using ∆∆C_t_ value for each amplified patient sample and cDNA from a healthy control. Data were analyzed by Applied BiosystemStepOne software v2.1 [12].

Statistical Analysis

Data were presented as mean±SD. Comparison between groups was performed by non-parametric Kruskal-Wallis and one-way ANOVA tests. A p value <0.05 was considered statistically significant. 

## RESULTS

Demographic and basic characteristics of studied groups are shown in Table 1. Regarding the percentage of NK cell subsets in lymphocyte population, CAD group presenting higher percentages of NK CD56^bright^ cells compared to the WFG recipients (p<0.05). However, no difference was observed in NK CD56^dim^ cells frequency or CD56^dim^/CD56^bright^ ratio between the two groups (Table 2).

**Table 1:** Patients’ demographics and clinical data: patients with chronic allograft dysfunction (CAD) and patients with well-function graft (WFG)

Parameter	Group	
	CAD (n=30)	WFG (n=30)
Mean±SD age in (yrs)	39.4±13.3	39.1±12.3
Male:Female	19:11	19:11
Cadaver/living donor	15:15	17:13
Mean±SD Cockcroft creatinine clearance (mL/min)	34.4±13.1	96.3±14.1
Mean±SD time post-transplantation (months)	41.0±17.8	41.0±17.8
IS protocol:*		
CsA, MMF, Steroids	22 (73%)	26 (87%)
Tac, Aza, Steroids	5 (17%)	3 (10%)
Rapa, MMF, Steroids	3 (10%)	1 (3%)

* IS: immunosuppressive, Aza: azathioprine, CsA: cyclosporine-A, MMF: mycophenolate mofetil, Tac: tacrolimus

**Table 2:** Frequency of NK CD56^dim^ and NK CD56^bright^ cells in CAD and WFG recipients

	CAD (n=30)	WFG (n=30)	p value
NK56^dim^ (%)	7.7±3.4	7.0±3.8	0.375
NK56^bright^ (%)	1.3±1.8	0.9±1.5	0.039
NK56^dim^/NK56^bright^	15.0±15.8	18.5±31.4	0.508

Frequency of NK CD56^dim^ and NK CD56^bright^ cells and NK CD56^dim^/NK CD56^bright^ ratio were also evaluated in CAD patients with different pathological grades of IF/TA. Despite a mild decrease in NK CD56^dim^ cell percentage and a subtle rise of NK CD56^bright^ among recipients with advanced pathology, there was no significant difference between CAD patients with different grades of IF/TA (Table 3). 

**Table 3:** Frequency of NK CD56^dim^ and NK CD56^bright^ cells in different grades of interstitial fibrosis/tubular atrophy

	Grade I (n=8)	Grade II (n=16)	Grade III (n=6)	p value
NK56^dim^ (%)	8.2±3.0	7.8±4.0	6.8±2.4	0.223
NK56^bright^ (%)	0.6±0.5	1.8±2.3	1.2±0.9	0.280
NK56^dim^/NK56^bright^	20.8±16.2	11.9±14.2	16.3±19.8	0.678

Comparison of IL-2, IL-15, and IL-18 genes expression between the studied groups showed no significant variation between patients with CAD and WFG (Table 4).

**Table 4:** Gene expression of interleukins 2, 15, and 18 in CAD and WFG patients

	CAD (n=30)	WFG (n=30)	p value
IL-2	0.04±0.14	0.13±0.30	0.163
IL-15	34.4±97.30	5.81±17.71	0.506
IL-18	6.46±12.85	2.67±3.00	0.871

According to the inclusion criteria set, none of patients had infectious diseases at time of sampling. However, a number of patients had past medical history of CMV positivity (IgM and IgG antibody to CMV). Therefore, the correlation between CMV positivity history and NK cells subsets frequency was evaluated in these patients. NK CD56^dim^ cells level was significantly higher among WFG patients with history of CMV positivity compared with CMV-negative patients (p=0.025) (Table 5).

**Table 5:** Frequency of NK CD56^dim^ and NK CD56^bright^ cells in CAD and WFG groups according to CMV antigen positivity

	CAD (n=30)			WFG (n=30)		
	CMV^–^(n=20)	CMV^+^ (n=10)	p value	CMV^– ^(n=23)	CMV^+^ (n=7)	p value
NK56^dim^ (%)	8.26±3.53	6.66±3.08	0.109	6.21±3.88	9.67±2.23	0.025
NK56^bright^ (%)	1.27±1.65	1.52±2.20	0.713	0.90±1.62	0.79±0.84	0.783
NK^dim^/NK^bright^	18.08±18.26	9.04±7.00	0.542	19.63±35.99	15.10±11.43	0.266

No significant difference was observed in NK CD56^dim^ and NK CD56^bright^ cell percentages among renal allograft recipients receiving different immunosuppressive regimes including cyclosporine (Sandimmune), tacrolimus, and rapamycin.

## DISCUSSION

NK cells, an important part of innate immunity, play a critical role in host defense against intracellular microorganisms and tumor cells by exerting cytotoxic activity and producing cytokines like INF-γ [13]. The NK cells controversial role in transplantation has been also considered in order to find a way to modulate their various activities for reaching better allograft survival [14].

Some of well-known disadvantages of presence of NK cells in allograft are killing donor cells according to the “missing self” theory [1], activating T cells via INF-γ secretion and OX40-OX40L interaction [15, 16], contribution to antibody-mediated injuries by antibody-dependent cytotoxicity (ADCC) mechanism [17], and predisposing endothelial damage because of MICA recognition by NKG2D on NK cells, which results in enhanced cytotoxicity [18, 19].

On the other hand, there are studies in favor of NK cells positive effects on allograft. For instance, Laffont, *et al*, showed that NK cells can prevent T cells direct activation by killing donor APCs [20]. Rabinovich, *et al*, showed perforin-mediated killing of T cells by NK cells [21]. Recent studies also indicate involvement of these cells in CD4^+^ and CD8^+^ T cell regulation [22, 23]. The role of NK cells in inducing chronic allograft vasculopathy has also been shown in a study [24]. Furthermore, new findings suggest therapeutic modalities that interfere with NK cells function in order to selectively modulate their cytolytic activity against donor APCs and help inducing tolerance, especially in highly immunogenic transplants [6].

In the present study, we investigated NK cell subsets in two sex- and age-matched groups of renal allograft recipients with different transplantation outcomes within similar post-transplantation intervals. Regarding the two major peripheral NK cell subsets, the CD56^bright^ NK cell subset was found to be significantly increased in CAD patients in comparison with WFG patients. However, there was a similar percentage of NK CD56^dim^ in studied groups. Although this finding can be partially explained by the effect of immunosuppressive drugs on development of NK cells, other probable underlying causes have to be investigated.

We also analyzed NK CD56^dim^/NK CD56^bright^ cells ratio, and found that it was also similar in WFG and CAD recipients. We could not find any association between advanced and mild pathological grades of IF/TA and NK subsets percentage.

There are evidence for the effect of various cytokines on NK cell maturation and activation; for example, IL-2 helps NK CD^bright^ cells to produce INF-γ [25], and IL-1, IL-2, IL-12, IL-15 and IL-18 contribute to NK CD^dim^ cells cytolytic activity [26, 27]. IL-2 and IL-12 cytokines genes polymorphism have been investigated in various immunologic disorders [28, 29]. Therefore, we evaluated gene expression of a cytokine profile to find any differences between WFG recipients and patients with CAD. Nevertheless, IL-2, IL-15, and IL-18 genes represented similar ranges of expression in these groups.

There are also studies indicating inhibitory effect of cyclosporine-A on NK cells global activation [30]. Some researchers showed that cyclosporine-A reduces the number of NK CD56^dim^ cells and causes a significant increase in NK CD56^bright^ subset frequency [31, 32]. However, considering prior evidence about this issue, we did not find any significant correlation between immunosuppressive regimens and peripheral NK cell repertoire of kidney transplant recipients, probably because of low study power. 

In conclusion, the present study demonstrated a significant increase in NK CD56^bright^ cells among CAD patients with biopsy-proven IF/TA lesions without considerable alteration in related cytokines gene expression in comparison with WFG recipients, suggesting a possible defect in NK cell maturation in these patients. Nonetheless, larger detailed studies are required to further assess the function and relevance of NK cells in renal transplantation long-term outcomes.
